# Care-seeking behaviour of caregivers for suspected malaria in under-five children in a Southwestern State of Nigeria

**DOI:** 10.1186/s12936-025-05433-3

**Published:** 2025-10-06

**Authors:** Omotola T. Akinrinade, Elvis E. Isere, IkeOluwapo O. Ajayi

**Affiliations:** 1 Department of Epidemiology and Medical Statistics, University of Ibadan, Ibadan, Nigeria; 2 Datamatrics Associates Ltd, Abuja, Nigeria

**Keywords:** Malaria, Care seeking behaviour, Caregivers, Under-five children, Nigeria

## Abstract

**Background:**

Effective malaria control in under-five children depends on caregivers seeking timely medical care. However, despite available healthcare services, many caregivers do not utilize health facilities, undermining malaria control efforts. This study assessed care-seeking behaviour of caregivers for suspected malaria in under-five children and associated factors in a southwest state of Nigeria.

**Methods:**

A cross-sectional analytical study was conducted in Ondo State, Southwestern Nigeria. The study areas were Ifedore, Ondo East, and Idanre Local Government Areas (LGAs) randomly selected from the eighteen LGAs in Ondo state. A multistage sampling technique was used to select participants and data was collected using a structured questionnaire. Descriptive and inferential statistics were applied to analyse the data, with logistic regression identifying significant predictors of care-seeking behaviour of caregivers of under-five children at p-value < 0.05.

**Results:**

The study included 301 caregivers of under-five children, predominantly aged 26–45 years (69.4%), males were 50.8% compared to females (49.2%) and 66.1% residing in rural areas. While 84.4% of caregivers identified infected mosquito bites as the cause of malaria, and 98.3% recognized fever as a main symptom, only 51.2% demonstrated good overall knowledge of malaria and symptoms in under-five children. Although 94.0% of caregivers suspected malaria in their under-five children within 12 months prior to the study, however, only 9.3% visited a health facility for treatment of their under-five children. In contrast, 37.4% purchased drugs from patent medicine vendors and pharmacies, while 25.2% used herbs for home management. Bivariate analysis showed significant associations between health facility visits and being a female caregiver (p = 0.009), urban residence (p = 0.002), residing within 5 km of a facility (p = 0.021), and good malaria knowledge (p = 0.033). Multivariate logistic regression indicated that female caregivers (aOR = 3.32, 95% CI 1.29–8.54), urban residents (aOR = 4.25, 95% CI 1.72–10.48), residing within 5 km of a health facility (aOR = 3.38, 95% CI 1.03–11.07), and those with good malaria knowledge (aOR = 4.16, 95% CI 1.61–10.77) were significant predictors of visiting a health facility for malaria care for under-five children with suspected malaria by caregivers.

**Conclusion:**

The study revealed low utilization of health facilities by caregivers seeking malaria treatment for under-five children with suspected malaria. Therefore, targeted community awareness campaigns are recommended to encourage caregivers to seek prompt, facility-based malaria care for under five children. Furthermore, in hard-to-reach or underserved rural areas with limited access to healthcare services, training and sensitization programmes for caregivers on appropriate home-based management of malaria including the use of pre-packaged artemisinin-based combination therapies are recommended.

## Background

Malaria is a life-threatening disease predominantly found in tropical regions [1–4]. It remains a major global public health concern, with sub-Saharan Africa recording the highest burden [1–3]. Among the most vulnerable populations, children under-five experience a disproportionate share of malaria-related morbidity and mortality [1–3].

Nigeria has the highest malaria burden globally, with the disease being a leading cause of childhood mortality [5]. According to the 2023 World Malaria Report, Nigeria recorded over 66 million malaria cases in 2022, accounting for 27% of global malaria cases and 26.8% of global malaria deaths, with children under-five being the most affected [6].

Care-seeking behaviour among caregivers plays a crucial role in the timely management of childhood illnesses, including malaria [7–10]. Early and appropriate treatment depends on caregivers' ability to recognize symptoms, their perception of illness severity, and their access to healthcare services [1, 7, 9, 11, 12]. More importantly, malaria treatment outcomes in children under-five are closely linked to caregivers' health-seeking behaviour [13].

Previous research on malaria care-seeking behaviour among caregivers in Nigeria highlights key trends and gaps [14–16]. Studies have indicated that caregivers of under-five children are pivotal in determining whether children receive timely health facility malaria treatment [14–16]. However, delayed healthcare-seeking in health facilities remains a major concern in Nigeria [14–16]. Several factors influence these behaviours, including the availability and accessibility of healthcare facilities, caregivers’ knowledge and perceptions of malaria, and sociocultural beliefs regarding under-five malaria treatment among households [14–16].

Regional disparities in healthcare access and cultural influences further shape malaria care-seeking behaviour in Nigeria [14–16]. In the southwest region, for instance, differences in healthcare infrastructure, economic factors, and traditional beliefs may influence caregivers' decisions regarding malaria treatment for under-five children [14–16]. Understanding these determinants is essential for developing targeted interventions that improve access to prompt and effective malaria care for children under-five at health facilities [14–16].

This study aims to investigate healthcare-seeking behaviour for suspected malaria in under-five children and associated factors among caregivers in Ondo state, southwestern Nigeria. Insights from this research will contribute to evidence-based policies and programmes designed to address region-specific caregivers’ behavioural barriers to accessing health facility malaria treatment for under-five children and improve child health outcomes.

## Methods

### Study area

Nigeria is the most populous country in Africa, with an estimated population of over 220 million [17, 18]. It has six geographical zones with varying ecologies, climates, and population characteristics. The 36 states are divided into zones, and the Federal Capital Territory, is further divided into 774 local government areas (LGAs) or districts and 8812 administrative wards [19].

**Fig. 1:**
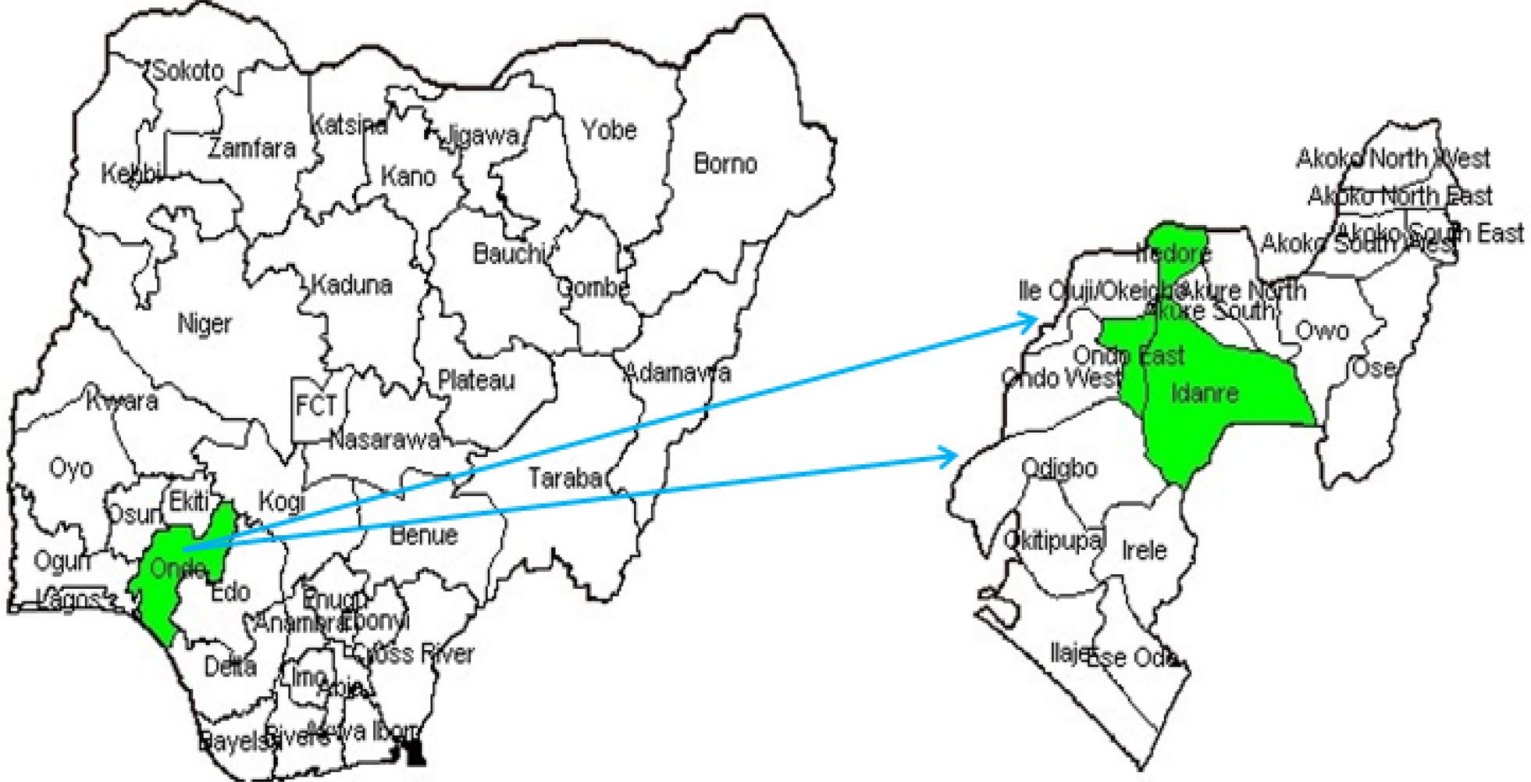
Map of Nigeria showing Ondo State and the study areas

Ondo state, where the study was conducted, is located in Southwestern Nigeria (Fig. 1). The state has 18 LGAs with three senatorial districts, including Ondo North, Central, and South with 2024 projected populations of about 5,858,113 based on the 2006 population census [18]. The state has about 800 primary health facilities, 18 general hospitals, 6 tertiary health facilities, and several private health facilities located across all LGAs in the state [20].

A significant proportion of the population in Ondo state are farmers engaged in the cultivation of both food and cash crops on a small-scale basis [21]. Additionally, hunting and livestock rearing, particularly goats, sheep, and fish, are prevalent occupations. The state's economic landscape also encompasses trading, civil service, and various government employment opportunities [22].

### Study design

A cross-sectional analytical study was conducted using a questionnaire survey method.

### Study population

The study participants were caregivers of under-five children in households randomly selected from settlements in three LGAs: Ifedore, Ondo East and Idanre LGAs of Ondo State.

### Eligibility criteria

#### Inclusion criteria

Caregivers of under-five children (biological parents, guardians, relatives, or other primary caregivers who provide essential care, such as feeding, hygiene, healthcare-seeking, and general welfare for under-five children within a household), who were 18 years and above, must have resided in the settlement in the last 12 months to the study and voluntarily consent to participate in the study.

#### Exclusion criteria

Selected households with caregivers of under-five children were excluded if they met the inclusion criteria, but the caregiver was absent at the time of the visit to the household or they refused to give informed consent to participate in the study. Additionally, caregivers with diagnosed or known mental illness or cognitive limitations were excluded from the study to ensure the accuracy and reliability of self-reported data on care-seeking behaviours. These conditions may impair decision-making, recall ability, and comprehension of survey questions, which could introduce bias and affect the validity of the findings.

### Sample size estimation

To determine the sample size for the study, Kish and Leslie’s formula [22] for estimating single proportions and estimation for minimum sample size was applied, resulting in an estimated sample size of 233 and 10% contingency for non-response, assuming a design effect of 1.5. Kish and Leslie’s formula: n = Z^2^P (1–P)/d^2^ where: n = sample size; Z = standard deviation for a 95% confidence level (Z = 1.96); P = proportion of caregivers of under-five children with good malaria health seeking behaviour; d = acceptable difference (if 5%, d = 0.05); q = 1–p. The prevalence estimate was obtained from a previous study where 18.6% of caregivers utilized health facilities for malaria treatment of their under-five children in Imo state, Nigeria [9].

### Sampling method

A multistage sampling technique was employed in this study. In stage 1, we divided the 18 LGAs into three categories using the senatorial districts (6 LGAs in Ondo North senatorial district, 6 LGAs in Ondo South senatorial district, and 6 LGAs in Ondo Central senatorial district), from which one senatorial district (Ondo Central senatorial district) was randomly selected using balloting.

In stage 2, 3 LGAs were randomly selected (Idanre, Ifedore, and Ondo East) through balloting from a list of 6 LGAs (Akure South, Akure North, Idanre, Ifedore, Ondo East, and Ondo West) in Ondo Central senatorial districts.

In stage 3, 3 wards were selected from the list of wards in each selected LGA using a simple random technique (balloting).

In stage 4, 2 settlements out of an average of 30 settlements per ward were sampled using a simple random technique (balloting). A total of 18 settlements were randomly selected from the list of settlements generated from each LGA.

In stage 5, households were sampled from each selected settlement using a spatial systematic random sampling with a random start technique. The middle of each settlement was identified, and then a pen was tossed. Sampling started from the house in the direction of the tip of the pen. For settlements with less than 20 houses, one house was skipped, while two houses were skipped for settlements with more than 20 houses. Sampling continued until the required sampling size expected for the settlement was reached [21, 23].

Finally, in stage 6, eligible caregivers of under-five children in each selected household who met the inclusion criteria were interviewed. A household was defined as a group of people living together daily in the same house and sharing meals from the same pot [21].

### Data collection

Caregivers of households with under-five children were interviewed by trained research assistants (RAs) using a pre-tested structured interviewer-administered questionnaire after signing the informed consent form. Research assistants were trained hands-on for 2 days prior to field data collection on the overview of malaria including the cause, symptoms and treatment, interviewing technique, ethical principles guiding health data collection, data collection using paper-based questionnaires, and addressing issues from community surveys. Field practical training was conducted on the third day of the training. Thereafter research assistants proceeded to the field for data collection which spanned for 10 days. Data were collected using paper-based questionnaires. Information collected were socio-demographic characteristics, knowledge of the cause and symptoms of malaria in under-five and malaria care-seeking behaviour after suspecting malaria infection in their under-five children. The supervision of data collection was done by the principal investigator.

### Data analysis

The data obtained were entered into a secured file structure on IBM Statistical Package for Social Sciences version 25.0 and analysed using frequency counts, percentages, cross tabulations, and logistic regression analysis with p < 0.05 level of significance. For knowledge score of malaria and symptoms in under-five children among caregivers, respondents were scored using a 15-item question. A point was assigned to the correct response to each question. The total score of each participant was divided by the total expected score (15 points) and multiplied by 100 to derive the percentage score. A modified Bloom’s cut-off was adapted to classify knowledge levels, using a 60% threshold as the distinction between good and poor knowledge. Bloom’s taxonomy is widely used in educational and health research to categorize learning and comprehension levels (Bloom). The adaptation of a 60% threshold is based on prior studies in health literacy, where similar cut-offs have been applied to differentiate between good and poor knowledge levels [24].

The association of under-five caregivers’ demographic characteristics and knowledge of malaria in under-five children with the care seeking behaviour for under-five malaria treatment was evaluated using the Chi-square test. In addition, unadjusted odds ratio (OR) and 95% confidence interval (CI) were estimated for caregivers’ malaria care seeking behaviour for under five children. A multivariate logistic regression was built to assess the association between the study outcome (visiting health facilities for malaria treatment of under five = 1 and visiting other places/interventions = 0), and all other variables that were considered to be significantly associated with the study outcome at the bivariate level (p < 0.05). The final model was interpreted using adjusted odds ratio (AOR) and corresponding 95% CI.

### Ethical consideration

Ethical approval was obtained from the Ondo State Health Research Ethics Committee of the Ondo State Ministry of Health, Akure, Nigeria with protocol number: AD. 4693 Vol. II/41. Also, Informed consent was obtained from the respondents. Before data collection, respondents were approached individually by trained research assistants and given a detailed explanation of the study. The research assistants introduced themselves, explained the study’s objectives, and ensured that each respondent understood the purpose of their participation. The explanation was provided in a language (English, Yoruba, or Pidgin) that the respondents were comfortable with to enhance comprehension. Respondents were informed that participation was entirely voluntary, meaning they had the right to refuse or withdraw at any time without facing any consequences. They were reassured that their decision would not affect their access to healthcare services or other benefits of the study.

To formalize consent, respondents were provided with an Informed Consent Form outlining the study’s purpose, procedures, potential risks, benefits, and confidentiality measures. If literate, they were asked to read and sign the form. If illiterate, a witness who could read and write was asked to interpret the content of the form to the respondent, after which the respondent provided consent through a thumbprint, witnessed by the interpreter. Signed or thumbprinted forms were securely stored as proof of consent before proceeding with data collection. Data collected from respondents were kept confidential using a password ODK secured database with access provided only to the principal investigator.

## Results

A total of 301 caregivers of under-five children were recruited for the study with more than half of the respondents between 26–45 years old (69.4%), 21.3% in the age group 18–25 and 9.3% in the age group 46 or older (Table 1**)**. The gender distribution among the respondents indicates a nearly equal distribution of male; 50.8% and female; 49.2% caregivers. Respondents are evenly distributed across the three LGAs (Idanre, 32.2%; Ifedore, 33.9%; Ondo East, 33.9%), each having around one-third of the respondents. About two-thirds of the respondents resides in rural areas (66.1%) compared to urban areas (33.9%). Respondents were predominantly Yoruba (62.5%), followed by Igbo (21.3%), Hausa (9.3%), and other ethnic groups (7.0%). Almost half had secondary education (42.9%), followed by tertiary education (29.9%), primary education (19.3%), and no formal education (8.0%).

**Table 1:** Characteristics of respondents and their households (N = 301)

Variables	Frequency, n (%)
Age group of caregivers (years)	
18–25	64 (21.3)
26–45	209 (69.4)
≥ 46	28 (9.3)
Gender of caregiver	
Female	148 (49.2)
Male	153 (50.8)
LGA of residence	
Idanre	97 (32.2)
Ifedore	102 (33.9)
Ondo East	102 (33.9)
Settlement type	
Urban	102 (33.9)
Rural	199 (66.1)
Ethnicity	
Others^a^	21 (7.0)
Hausa	28 (9.3)
Igbo	64 (21.3)
Yoruba	185 (62.5)
Marital Status of caregiver	
Single	24 (8.0)
Separated/Divorce/Widowed	39 (13.0)
Married	238 (79.1)
Educational level of caregivers	
No formal education	24 (8.0)
Primary	58 (19.3)
Tertiary	90 (29.9)
Secondary	129 (42.9)
Occupation	
Unemployed	26 (8.6)
Private organization	38 (12.6)
Farming	59 (19.6)
Civil servant	68 (22.6)
Trading	110 (36.5)
Religion	
Traditional worshipper	14 (4.7)
Islam	69 (22.9)
Christianity	218 (72.4)

Others^a^: Ijaw, Urhobo, Tiv

From Table 2, about two-thirds of the respondents live within 5 kms of a health facility (67.1%), with others living ≥ 5 kms away (32.9%). Similarly, more than half of the respondents would reach the nearest health facilities within 30 min (57.5%). Others take more than 30 min (42.5%) irrespective of the mode of transportation. The most common mode of transportation to health facilities is public taxis (42.5%), followed by walking by foot (38.2%), and using private vehicles (19.3%).

**Table 2:** Other characteristics of respondents and their household (n = 301)

Variable	Frequency, n (%)2
Age group of child (months)	
< 10	69 (22.9)
10–29	107 (35.5)
30–59	125 (41.5)
Gender of child	
Female	135 (33.9)
Male	166 (55.1)
Number of household members	
< 5	84 (27.9)
5–9	174 (57.8)
≥ 10	43 (14.3)
Transportation mode to health facility	
Private vehicle	58 (19.3)
On foot	115 (38.2)
Public taxi	128 (42.5)
Time taken to reach the nearest health facility irrespective of mode of transportation (minutes)	
≥ 30 min	128 (42.5)
< 30 min	173 (57.5)
Distance to nearest health facility	
≥ 5 km	99 (32.9)
< 5 km	202 (67.1)

Table 3 shows the caregiver’s knowledge of malaria and symptoms in under-five children. A vast majority of respondents (84.4%) attributed malaria to infected mosquito bites. Fever was also the most widely recognized symptom (98.3%), followed by headache (94.4%), loss of appetite (87.4%). More than two-thirds of the respondents (74.8%) considered visiting health facilities as the most appropriate source of treatment. Other common practices mentioned by the respondents were buying malaria drugs from vendors (71.4%), visiting traditionalists or spiritualists for prayers (56.5%), and taking herbal concoctions at home (49.8%). Overall, just about half (51.2%) had good knowledge of malaria and symptoms in under-five children compared to 48.8% who had poor knowledge.

**Table 3:** Knowledge of malaria and symptoms in under-five children among respondents

Variable	Frequency, n (%), *N* = 301	
	Yes	No
Causes ofmalaria		
Infected mosquito bite	254 (84.4)	47 (15.6)
Spiritual gods/attack	110 (36.5)	191 (63.5)
Common symptoms of malaria		
Bleeding	62 (20.6)	239 (79.4)
Stooling	122 (40.5)	179 (59.5)
Backache	169 (56.1)	132 (43.9)
Loss of weight	206 (68.4)	95 (31.6)
Vomiting	215 (71.4)	86 (28.6)
Body weakness	236 (78.4)	65 (21.6)
Loss of appetite	263 (87.4)	38 (12.6)
Headache	284 (94.4)	17 (5.6)
Most appropriate way of treating malaria		
Taking herbal concoction	150 (49.8)	151 (50.2)
Visiting a traditionalist or spiritualist for prayers	170 (56.5)	131 (43.5)
Buying malaria drugs from vendors	215 (71.4)	86 (28.6)
Visiting health facilities for treatment	225 (74.8)	76 (25.2)

In the last 12 months, 94.0% of caregivers reported that their child has had malaria related symptoms as shown in Table 4**.** However, among those whose children experienced symptoms, only 9.3% visited a health facility for treatment (Table 4). Among caregivers who did not visit a health facility (n = 255), the main reasons were indecision by caregiver on where to seek care for their child(51.8%) and lack of trust in health facility care services and healthcare workers(19.2%).

**Table 4:** Care-seeking behaviour for suspected malaria in under-five by caregivers

Variable	Frequency, n (%), N = 301
Has your child had malaria related symptoms in the last 12 months	
No	18 (6.0)
Yes	283 (94.0)
How did you treat your child	n = 283
Visited a traditional healer for treatment	11 (3.2)
Visited a spiritualist with child for prayers	25 (8.3)
Visited a health facility immediately	28 (9.3)
Home rest without medicine	29 (9.6)
Treated the child with herbs at home	76 (25.2)
Visited a patent medicine vendor/pharmacy for treatment	114 (37.4)
Reasons for not visiting a health facility immediately you noticed the symptoms of malaria in your child?	n = 255
Long distance to clinic	3 (1.2)
Attitude of health workers	4 (1.6)
Transportation cost	27 (10.6)
Cannot leave work (overlapping work hours with health facility working hours)	40 (15.7)
Lack of trust in health facility care services and healthcare workers	49 (19.2)
Caregiver indecision on where to seek health care for child	132 (51.8)

Table 5 shows the results of bivariate analysis. Female caregivers were significantly more likely to visit health facilities for malaria treatment of under-five children (14.7%) compared to male caregivers (5.4%) (X^2^ = 6.800, p = 0.009). Similarly, caregivers in urban settlements had a significantly higher likelihood of visiting health facilities (17.7%) for malaria treatment of under five children compared to those in rural areas (5.9%) (X^2^ = 9.951, p = 0.002). Although caregivers with tertiary education showed a higher propensity to visit health facilities (12.9%) for malaria treatment of under five children relative to those with no formal education (4.3%), primary education (14.3%), or secondary education (6.7%), these differences were not statistically significant (X^2^ = 4.233, p = 0.237). The analysis also indicated that caregivers who took less than 30 min to access a health facility were significantly more likely to visit health facilities for the treatment of under-five children for malaria (13.1%) compared to those who took 30 min or more (5.7%) (X^2^ = 4.233, p = 0.038). Additionally, caregivers residing within 5 km of a health facility were significantly more likely to seek malaria treatment (12.8%) in health facility compared to those living 5 km or more away (4.2%) (X^2^ = 5.346, p = 0.021). Lastly, caregivers with good knowledge of malaria and symptoms were significantly more likely to utilize health facilities (13.8%) for malaria treatment of under-five children compared to those with poor knowledge (6.2%) (X^2^ = 4.534, p = 0.033).

**Table 5:** Association of care-seeking behaviour for suspected malaria with respondent’s characteristics’

Variable	Health-seeking behaviour and malaria treatment practice		Total, N (%)	X^2^	p-value†
	Visited health facility for malaria treatment, n (%)	Visited others places to seek malaria treatment, n (%)			
Age group of caregivers (years)					0.325
18–25	9 (15.0)	51 (85.0)	60 (100)	2.249	
26–45	17 (8.6)	180 (91.4)	197 (100)		
≥ 46	2 (7.7)	24 (92.3)	26 (100)		
Gender of caregiver					
Male	8 (5.4)	139 (94.6)	147 (100)	6.800	0.009*
Female	20 (14.7)	116 (85.3)	136 (100)		
Settlement type					
Rural	11 (5.9)	176 (94.1)	187 (100)	9.951	0.002*
Urban	17 (17.7)	79 (82.3)	96 (100)		
Ethnicity					
Yoruba	22 (12.4)	155 (87.6)	177 (100)	3.407	0.065
Others^a^	6 (5.7)	100 (94.3)	106 (100)		
Educational level of caregivers					
No formal education	1 (4.3)	22 (95.7)	23 (100)	4.233	0.237
Primary	8 (14.3)	48 (85.7)	56 (100)		
Secondary	8 (6.7)	111 (93.3)	119 (100)		
Tertiary	11 (12.9)	74 (87.1)	85 (100)		
Occupation					
Unemployed	3 (12.0)	22 (88.0)	25 (100)	6.709	0.152
Civil servant	10 (15.4)	55 (84.6)	65 (100)		
Farming	3 (5.8)	49 (94.2)	52 (100)		
Trading	6 (5.8)	97 (94.2)	103 (100)		
Private organization	6 (15.8)	32 (84.2)	38 (100)		
Religion					
Christianity	22 (10.6)	186 (89.4)	208 (100)	0.411	0.522
Other religion	6 (8.0)	69 (92.0)	75 (100)		
Number of household members					
< 5	12 (15.0)	68 (85.0)	80 (100)	3.527	0.171
5–10	14 (8.4)	153 (91.6)	167 (100)		
≥ 10	2 (5.6)	34 (94.4)	36 (100)		
Time taken to access health facility irrespective of mode of transportation (minutes)					
< 30 min	21 (13.1)	139 (86.9)	160 (100)	4.311	0.038*
≥ 30 min	7 (5.7)	116 (94.3)	123 (100)		
Distance to nearest health facility					
< 5 km	24 (12.8)	163 (87.3)	187 (100)	5.346	0.021*
≥ 5 km	4 (4.2)	92 (95.8)	96 (100)		
Knowledge of Malaria causes and symptoms					
Good knowledge	19 (13.8)	119 (86.2)	138 (100)	4.534	0.033*
Poor Knowledge	9 (6.2)	136 (93.8)	145 (100)		

Others^a^: Ijaw, Urhobo, Tiv, Igbo, Hausa, † Chi square test of association, *Significance at p < 0.05

Multivariate logistic regression analysis revealed that being a female (AOR = 3.32, 95% CI 1.29–8.54), residing in an urban settlement (AOR: 4.25, 95% CI 1.72–10.48), residing at a distance less than 5 km from a health facility (AOR = 3.38, 95% CI:1.03 −11.07) and having good knowledge of malaria and symptoms in under-five children (AOR = 4.16, 95% CI 1.61–10.77) were significant predictors of seeking care for suspected malaria in under-five children at health facility by caregivers (Table 6**).**

**Table 6:** Predictors of care-seeking behaviour for suspected malaria in under-five children by caregivers

Variables	Care-seeking behaviour for suspected malaria			
	Unadjusted Odds Ratio (95% CI)	p-value	Adjusted Odds Ratio (95% CI)	p-value
Sex				
Male	R		R	
Female	2.99 (1.27–7.05)	0.012	3.32 (1.29–8.54)	0.013
Settlement type				
Rural	R		R	
Urban	3.44 (1.54–7.69)	0.003	4.25 (1.72–10.48)	0.002
Time taken to access health facility (minutes)				
≥ 30 min	R		R	
< 30 min	2.50 (1.03–6.09)	0.043	2.43 (0.90–6.56)	0.080
Distance to nearest health facility				
≥ 5 km	R		R	
< 5 km	3.39 (1.14–10.06)	0.028	3.38 (1.03–11.07)	0.044
Knowledge of Malaria causes and symptoms				
Poor knowledge	R		R	
Good Knowledge	2.41 (1.05–5.53)	0.038	4.16 (1.61–10.77)	0.003

## Discussion

This study revealed that majority of caregivers of under-five children did not seek care from health facilities when their children had malaria related symptoms. Less than one-tenth of caregivers with under-five children who had symptoms of malaria in this study reported visiting a health facility for suspected malaria treatment. This finding aligns with previous studies which reported suboptimal utilization of health facility services among caregivers of children under-five seeking care and treatment for malaria in other regions of Nigeria [8, 9, 14, 25, 26]. Nwaneri and Sadoh [8] in a similar study in southeast Nigeria reported that only 29.0% of caregivers with children under-five sought malaria treatment from a health facility. Similarly, Oluchi et al. [9]*.* found that 18.6% of caregivers sought treatment from health facilities within 24 h of the onset of fever in their under-5 year old children.

Notably, over fifty percent of the respondents in this study had good knowledge about malaria and symptoms in under-five children indicating a good ability/capacity among the caregivers to promptly recognize malaria symptoms in under-five children. In addition, most of the respondents reported fever as a common symptom of malaria and mosquitoes as the cause of malaria infection while almost three quarters of the respondents knew visiting a health facility as the most appropriate care seeking practice for malaria treatment for under-five children. Interestingly, the high proportion of respondents that had good knowledge about malaria and symptoms in under-five children in this study could be attributed to the high education status of respondents as over two third of the respondents had at least secondary level of education and so could read and get general information about malaria. Similar studies conducted in other regions of Nigeria have reported good knowledge about malaria among caregivers of under-five children in Enugu and Anambra states of Nigeria [1, 27]. This could be as a result of the mass awareness and prevention campaigns against malaria in under five organized by government health agencies across the states due to the very high burden of malaria and the high morbidity and mortality rate recorded across states in Nigeria annually [4, 5, 28, 29].

Although a considerable proportion of respondents in this study demonstrated good knowledge of malaria in under-five children, many still reported seeking malaria treatment for their children at patent medicine stores, pharmacies, or through home management with herbal remedies, spiritualists, or traditional healers instead of health facilities. The primary reasons for seeking care outside health facility reported in this study were indecision on where to seek care, long waiting times, transportation costs, healthcare workers' attitudes, lack of trust in health services, and distance to health facilities. These factors identified in this study aligns with findings from previous studies [30, 31].

Andersen-Newman Framework for Health Services Utilization indicates that utilization of health services is influenced by various factors such as population and environmental characteristics [32]. The study outcome where the significant predictors of care-seeking behaviour for suspected malaria in under-five children in a health facility among caregivers were being a female as a caregiver, residing in a rural settlement and less than 5 km to a health facility and having good knowledge about malaria in under-five children are in agreement with the above framework [32] and other research findings [9, 33]. Previous studies documented that women were traditionally, the primary caregivers in many Nigerian households [11, 34], more involved in day-to-day childcare activities, and are thus more likely to observe when a child is unwell and thereafter take necessary actions, including seeking medical care [11, 34–36]. In addition, the Nigerian cultural norms and societal expectations often designate women as responsible for family health and childcare [11, 33–35].

Furthermore, in the past, men on the other hand were known to be more involved in providing financial support or working outside the home, leaving women to manage healthcare decisions [36, 37]. However, this study reported more male caregivers than females. This could be attributed to the suboptimal health facility care utilization for under-five children among caregivers in this study as shown from the multivariate analysis as males have been reported to having very poor health-seeking behaviour over time [38].

Additionally, Nigeria is a country with diverse socio-cultural and religious beliefs hence the diverse and more progressive urban settings might have fewer cultural barriers to accessing healthcare, such as gender roles or traditional beliefs [16, 37]. Cultural beliefs and practices in rural areas can hinder the use of modern healthcare services such as seeking health care for malaria treatment in health facilities as indicated in this study where a significant proportion of the respondents sought treatment from traditional healers, spiritualist and home treatment using herbs for malaria care of their under five children (Table 4) [37]. In this study, the caregivers residing at a distance of less than five kilometres to the health facility were more likely to seek care for malaria treatment for their under five children from health facilities. Proximity to health facilities plays a significant role in influencing malaria care-seeking behavior for children under five among caregivers in Nigeria. Previous studies have shown that caregivers who reside closer to health facilities are more likely to seek timely treatment for malaria in children, improving health outcomes [11, 39].

Findings from previous research conducted in Nigeria revealed that distance to healthcare significantly impacts whether caregivers seek formal healthcare or rely on self-medication and traditional remedies. Adamu and Ango [39] had reported similar findings from their study conducted among caregivers of under-five children who had malaria and other childhood illnesses in Sokoto state, Nigeria. Notably, in cases where health facilities are far, caregivers often delay seeking care, which exacerbates the severity of malaria symptoms in children, and increasing the risk of complications or mortality [40]. This finding might account for the significant proportion of our respondents (37.4%) who sought malaria treatment from patent medicine vendors and pharmacy for under five children as these drug outlets are sited within the communities and are easily accessible by caregivers of under five children [40, 41].

The outcome of this study which shows that knowledge of malaria is associated with seeking treatment at health facilities among caregivers for under-five children with suspected malaria aligns with a previous study which found that malaria knowledge could improve health-related behaviours independently of other factors [42, 43]. This observation could be due to the fact that early treatment relies on the prompt recognition of malaria symptoms within the household [42, 44]. It appears that caregivers' knowledge about any disease including malaria is crucial for improving health-seeking behaviour [42, 44].

More importantly, the utilization rate of health facility services reported in this study by caregivers of under-five children highlights the importance of home management of malaria, which is not only feasible but essential in settings in Nigeria where healthcare access is challenging due to far distance of the nearest health facilities to most households within the communities especially in rural settings [45]. This approach of supporting communities to operationalize home management of malaria for under-five children using recommended antimalarial drugs when malaria is perceived by caregivers could greatly reduce the reliance on unverified treatment sources and improve child survival rates in malaria-endemic countries like Nigeria. The Nigerian Malaria Home Management Guidelines emphasized equipping caregivers with the knowledge and tools to manage malaria at home, especially in areas where accessing health facility services is challenging due to distance or logistical barriers [46, 47]. By providing caregivers with access to pre-packaged artemisinin-based combinations, training on malaria symptom recognition, and when to seek further medical care, especially in severe cases, the home management strategy can bridge the care gap in areas with limited healthcare access [16, 47–50].

It is not surprising that educational attainment did not significantly influence care-seeking practices at health facilities for under-five children in this study. Ondo State, located in southwestern Nigeria, is known for having one of the highest literacy rates in the country [51, 52]. This aligns with the finding from this study, where more than half of the caregivers had at least a primary education. In a setting with high literacy levels, access to health information is widespread through multiple channels, including community health programs, media, and public health campaigns. Additionally, government-led initiatives such as the “abiye” and “agbebiye” projects implemented in Ondo state over the years and a good healthcare infrastructure such as the building of Mother and child hospitals across the state in the last few years especially in the urban areas may have contributed to a general awareness of the importance of facility-based care among caregivers in the state, making healthcare-seeking behaviour less dependent on formal educational attainment [53–55]. This suggests that other factors, such as proximity to health facilities, healthcare affordability, and cultural beliefs, may play a more significant role in influencing care-seeking behaviour than education alone.

Interestingly, the findings of this study, although conducted in the southwest region of Nigeria, can be generalized to other malaria-endemic regions of the country with similar socio-economic and healthcare access challenges. Thus, the insights drawn from this study could inform broader malaria control and care-seeking strategies in other similar settings across Nigeria.

This study has some limitations. Firstly, the care-seeking behaviour reported in this study was based on suspected malaria by caregivers thus some cases may not have been malaria. However, this would not have changed the behaviour of the caregivers because they thought their children were sick and needed to provide healthcare for their children. The study may also not be free of recall bias since it was a self-reported malaria care seeking behaviourial practices for a recall period of 12 months. However, trained interviewers were engaged for this study as well as a well-structured questionnaire was used for the data collection. Additionally, probing questions were asked to ensure correct responses were provided by the respondents during the interviews. Furthermore, the cross-sectional design of this study limits the study to descriptive and associative interpretations. However, the study provides relevant information that can help in making pertinent policies in health service access and delivery for under-five children in Nigeria.

## Conclusion

The study revealed low utilization of health facilities by caregivers seeking malaria treatment for under-five children with suspected malaria. Therefore, targeted community awareness campaigns are recommended to encourage caregivers to seek prompt, health facility-based malaria care for under-five children. Furthermore, in hard-to-reach or underserved rural areas with limited access to healthcare services, training and sensitization programmes for caregivers on appropriate home-based management of malaria for under-five children including the use of pre-packaged artemisinin-based combination therapies are recommended. This approach could discourage the use of unauthorized malaria treatment among caregivers of under-five children.

## Data Availability

The data sets used or analyzed during this study are available from the corresponding authors upon reasonable request.
